# The therapeutic alliance in cognitive-behavioral therapy for obsessive-compulsive disorder: A systematic review and meta-analysis

**DOI:** 10.3389/fpsyt.2022.951925

**Published:** 2022-09-06

**Authors:** Francesca Strappini, Valentina Socci, Angelo Maria Saliani, Giuseppe Grossi, Giulia D’Ari, Titti Damato, Nicole Pompili, Guido Alessandri, Francesco Mancini

**Affiliations:** ^1^Department of Psychology, Sapienza University of Rome, Rome, Italy; ^2^Department of Biotechnological and Applied Clinical Sciences, University of L’Aquila, Coppito, Italy; ^3^Association School of Cognitive Psychotherapy (APC-SPC), Rome, Italy; ^4^Department of Human Sciences, Guglielmo Marconi University, Rome, Italy

**Keywords:** OCD, therapeutic relationship, alliance-outcome association, working alliance, therapeutic alliance

## Abstract

**Background:**

The therapeutic alliance has been recognized as one of the most researched key elements of treatment across different therapeutic approaches and diagnostic domains. Despite its importance, our current understanding of its clinical relevance in patients with obsessive-compulsive disorder (OCD) is still debated. This study aimed to examine empirical evidence on the effect of alliance on treatment outcomes in Cognitive Behavioral Therapy (CBT) in patients with OCD in a systematic review and meta-analysis.

**Methods:**

Original peer-reviewed articles until March 2022 were included if they were (1) written in English; (2) included a clinical group with a current primary OCD diagnosis; (3) involved individual CBT; (4) used a validated therapeutic alliance scale that was related to the outcome measurement; (5) reported an effect size.

**Results:**

Thirteen studies were included, six of which contained sufficient statistical information to be included in the meta-analysis. A total of 897 patients took part in all reviewed studies. We found a modest effect of alliance on post-treatment outcome [*Tau*^2^ = −0.1562 (*C.I. 95%*: −0.2542 to −0.0582)].

**Discussion:**

The results show the existence of considerable variability and methodological inconsistencies across studies. We discuss the role of methodological factors that could account for this divergence, the research limitations, and the implications for current research.

**Systematic review registration:**

[https://osf.io/dxez5/?view_only=bc2deaa7f0794c8dbef440255b2d4b3b].

## Introduction

Obsessive-Compulsive Disorder (OCD) is a serious mental health condition characterized by recurrent and persistent thoughts, urges, or images that are experienced as highly disturbing and intrusive (obsessions) and/or stereotyped recurrent mental or physical behaviors aimed to ignore or neutralize them (compulsions) (1). OCD symptom domains typically include contamination obsession and washing/cleaning compulsion; obsession concerning responsibility for harm, injury, or bad luck and checking compulsion; unacceptable obsessional thoughts concerning sex, violence, or religion associated with mental neutralizing strategies; obsession about symmetry, completeness, and exactness and ordering compulsion (2). Given the complexity and heterogeneity of symptoms, several genetic, behavioral, and cognitive models have been proposed to explain the mechanisms behind this spectrum [e.g., (3–6)].

The estimated lifetime prevalence of the full disorder is approximately 2–3%, with most individuals with OCD being affected before their mid-twenties (7, 8). OCD shows a chronic course, and it is highly comorbid with anxiety disorders and major depressive disorder (9). In the absence of effective treatment, OCD results in significant distress, functional impairments in social and occupational functioning, and reduced quality of life. Therefore, it is considered a disabling mental health condition associated with significant personal and socio-economic costs (10).

A combined approach that includes cognitive therapy and behavioral therapy represents the currently recommended psychological treatment of choice for OCD (11), showing the highest degree of empirical support in meta-analytic investigations [e.g., (12–14)]. According to NICE guidelines, it is recommended a “stepped care” model, with increasing intensity of treatment according to clinical severity and complexity (2005). This treatment includes exposure with response prevention (EX/RP) with or without OCD-focused cognitive therapy (CT). EX/RP is a behavioral therapy that comprises the implementation of a series of in-session and between-session exposures that are planned and implemented through collaboration between patient and therapist. The treatment generally includes more or less prolonged exposure to obsessional triggers and procedures aimed at blocking rituals. Although the optimal frequency of sessions has not been defined, both intensive, which involves daily sessions over 1 month, and weekly sessions, have been proved effective in reducing symptoms (15, 16). Compared to medications alone, EX/RP protocol is more effective with a lower relapse rate (17). However, despite the effectiveness of such structured, evidence-based treatment, up to 18.7% of OCD patients will drop out prior to completion of treatment (18). Further, about 50% of OCD patients still complain about some residual symptoms even after successful treatment, with a negative impact on their quality of life (19–21). Indeed, a significant proportion of OCD patients receiving cognitive and behavioral therapies is subjected to post-treatment relapse, with an estimated full recovery rate of approximately 25% (22). Despite the undoubtedly high impact that CT and EX/RP exert on patients, there is still a significant degree of variability associated with treatment response. This variance cannot be fully explained by the effect of the specific treatment and probably needs to be accounted for by other variables. Therefore, identifying the complex factors associated with successful treatment outcomes is crucial to optimizing the delivery of evidence-based psychotherapeutic interventions.

In the last decades, research in the field of psychotherapy has increasingly focused on examining potential mechanisms of therapeutic change, aiming to identify predictors of treatment response. In this context, the therapeutic relationship variables received particular attention, especially in the operationalized construct of the alliance. From a transtheoretical perspective, the alliance can be defined as a collaborative stance between client and therapist, underpinned by three components: (a) consensus on therapeutic goals, (b) agreement on therapeutic tasks, and (c) a positive bond between client and therapist (23). The therapeutic alliance is historically defined as a “non-specific” interpersonal factor auxiliary to technical procedures that produce change (24). Cognitive-behavioral perspective emphasizes the collaborative nature of the therapeutic alliance. Within this framework, the alliance is conceptualized as a necessary but not sufficient therapeutic change factor (25, 26), allowing the creation of trust and safety conditions between patient and therapist that, in turn, facilitate the application of specific techniques.

Nevertheless, the therapeutic alliance is widely recognized as a crucial component of treatment across all psychotherapeutic approaches. Accordingly, a substantial number of empirical research have been addressed to explore the association between alliance and post-treatment outcomes. In this respect, the recent Third Interdivisional APA Task Force on Evidence-Based Relationships and Responsiveness synthesized empirical studies investigating the association between the therapeutic relationship and outcome and suggested alliance as a “demonstrably effective” ingredient of the therapeutic change process across treatments and diagnoses (27). On the whole, literature accumulated so far suggest that the alliance is moderately associated with treatment outcomes in a transdiagnostic way (28–33), yet its impact on process change has been sparsely investigated in specific disorders. Indeed, most experimental and meta-analytic studies estimate the alliance-outcome association by aggregating disorders and treatments. However, evidence suggesting potential differences across disorders also exists, with some diagnostic groups being more affected by therapeutic alliance than others (34, 35). For instance, the alliance seems to have less impact on severe anxiety disorders, substance abuse, and eating disorders than on other disorders, such as depression (27, 32, 36–38). Notably, investigating how the alliance works for specific disorders has been delineated as one of the key questions for future studies by the Third Interdivisional APA Task Force on Evidence-Based Relationships and Responsiveness (27).

In this respect, no systematic review to date has been specifically aimed at exploring the alliance-outcome relationship in individuals receiving Cognitive-Behavioral Therapy (CBT) for OCD.

Although OCD is no longer categorized as an anxiety disorder in the DSM based on significant diagnostic validators, it is important to highlight that a substantial overlap between OCD and the anxiety disorders is still acknowledged by many clinicians and researchers [e.g., (39)]. For this reason, some recent reviews pulled together OCD with disorders like post-traumatic stress disorder, generalized anxiety disorder, and social anxiety disorder. Specifically, two recent qualitative reviews on the role of therapeutic alliance in anxiety-related disorders (32, 33) added a contribution to this research area. In their critical review, Buchholz and Abramowitz (32) provided an overview of existing research on the alliance-outcome relationship in exposure therapy for anxiety-related disorders, including OCD. Results suggest a link between a strong alliance and symptom reduction in EX/RP therapy for OCD, with some evidence indicating that task and goal alliance dimensions, relative to the bond alliance, were the strongest predictors of post-treatment outcome, along with treatment adherence. Importantly, this critical literature review also revealed substantial methodological and conceptual differences among investigations, including alliance assessment tools, timing and perspective of the alliance assessment, and diagnoses (32). Accordingly, in a subsequent critical review (33), the alliance-outcome relationship in CBT for anxiety disorders was also found to differ significantly across the timing of the alliance assessment (e.g., early, middle, and late assessment in the course of therapy), perspectives of the alliance rater (e.g., patient, therapist or observer-rated alliance), and specific alliance dimensions. However, it is important to note that the alliance-outcome relationship in OCD was not the primary focus of investigation in these recent literature reviews; further, both reviews restricted their critical analysis to adult samples and face-to-face therapies, potentially limiting the generalizability of the results to younger individuals with OCD as well as electronically delivered treatments such as internet-based CBT (iCBT).

Collectively, recent findings suggest a role of the alliance, particularly task and goal alliance dimensions, in predicting post-treatment outcomes in anxiety-related disorders, including OCD. Crucially, the literature accumulated so far has produced mixed findings potentially due to significant methodological and conceptual differences among studies (32, 33). Therefore, the effective role of the therapeutic alliance as a change mechanism in CBT for individuals with OCD remains unclear. The present systematic review and meta-analysis aimed to synthesize the available empirical studies investigating the relationship between therapeutic alliance and post-treatment outcomes with CBT in patients with OCD. Further clarifying the impact of the therapeutic alliance in the psychotherapeutic approach for individuals with OCD could enrich our understanding of effective therapeutic change factors implementing evidence-based treatments for this diagnostic group.

## Materials and methods

**FIGURE 1:**
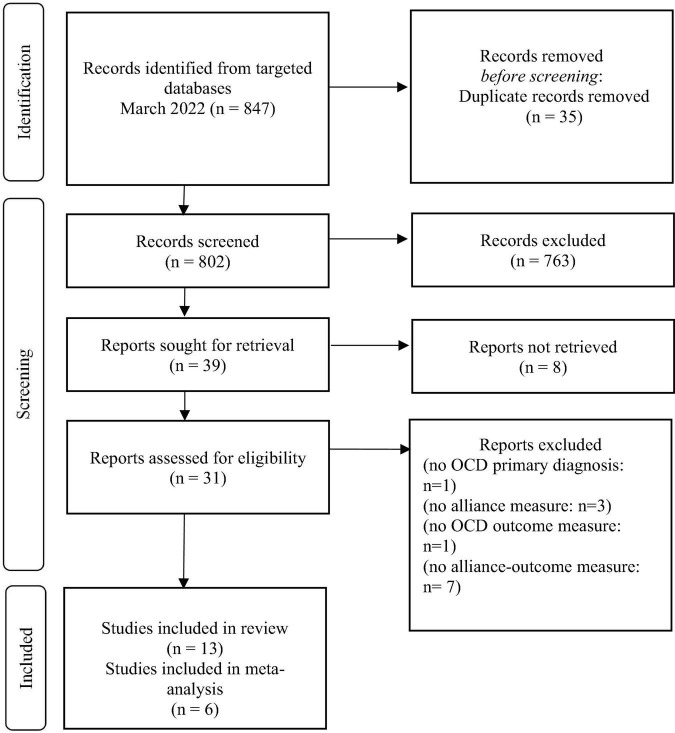
Flowchart of selection process for included articles.

The systematic review process was conducted according to the PRISMA guidelines (40–43)^1^ and preregistered on Open Science Framework (OSF).^2^ The PRISMA protocol consists of a 27-item checklist and a 4-phase flow diagram that guides the systematic review process (see Figure 1).

### Research strategies

We conducted a systematic search of articles published in peer-reviewed journals articled indexed in the following electronic databases: *PubMed* (1949 to March 2022), *Scopus* (1788 to March 2022), *PsycINFO* (1806 to March 2022), *PsychArticles* (1800 to March 2022), and *Web of Science* (1900 to March 2022). The search strategy used Boolean combinations of the following keywords: (“obsessive-compulsive disorder” OR “OCD”) AND (“therapeutic relationship” OR “working relationship” OR “collaborative relationship” OR “alliance” OR “working alliance” OR “helping alliance” OR “therapist factors” OR “mediator” OR “emotional bond” OR “alliance-outcome relationship”). Mendeley reference manager software^3^ was used to import the references from the databases and to remove duplicates. The first screening was made by reading the title and abstract by the authors F.S. and V.S. The same authors read the full text of the selected studies. In addition to systematic searches in the above databases, we also searched for additional articles in the reference lists of the selected papers (i.e., backward research) and identified studies that cited the selected articles (i.e., forward research).

### Eligibility criteria

According with our aims (i.e., investigating the relation between therapeutic alliance and outcome in OCD), we included studies that fulfilled the following criteria: (a) original, peer-reviewed articles; (b) written in English; (c) included a clinical group with a current primary OCD diagnosis; (d) were empirical and included quantitative data (i.e., reviews, case studies, and qualitative papers were excluded); (e) involved individual CBT; (f) used a validated therapeutic alliance scale with adequate psychometric properties (specifically, we included only studies that used the scales recommended by Martin et al. (31) and Elvins and Green (44) as core measures of the construct); (g) measured the relationship between therapeutic alliance and at least one systematic outcome measure (Yale-Brown Obsessive Compulsive Scale, Y-BOCS; Obsessive Compulsive Inventory, OCI) in the context of individual CBT for OCD; (h) a reported effect (*d* or *r*), its equivalent (standardized β weight), or other statistic (*t* or *F*) that could be converted to an effect. Articles from all publication years were accepted.

### Data collection

Descriptive and quantitative data extraction was performed from each study and included: (a) metadata (i.e., authors and year of publication); (b) information related to the sample (i.e., sample size, age, gender, and onset age); (c) methodological information (i.e., alliance and OCD scales, alliance and outcome rater(s), the timing of the assessment); (d) main results and effects size (see Table 1).

**TABLE 1:** Synthetic description of studies that have examined the influence of therapeutic alliance on treatment outcome.

Study	*N*	%*F*	Age	Onset (years)	Intervention	Outcome measure	OCD rater(s)	Alliance measure	Timing	Alliance rater(s)	Alliance-outcome relationship	Effect	Size	C.I. 95 %	For *r*
												r	d	LL	DL
Hoogduin et al. (79)	60				EX/RP	Self-monitoring	P	RI	Mid	P T	Yes	0.31 0.47	0.65 1.06	0.52 0.65	0.006 0.24
Hoogduin et al. (79)	25				EX/RP	Self-Monitoring	P	RI	Early Mid	P T	Yes	0.43 0.42	0.95 0.92	0.7 0.7	0.04 0.03
Keijsers et al. (63)	40P 9T	55	M = 34.8 SD = 13.7		EX/RP	MOCI	I.E.	RI	Early	P T	Yes	0.02 0.44	0.04 0.98	0.4 0.71	−0.38 0.05
Vogel et al. (76)	37	73	M = 35.1 SD = 12.1		EX/RP	Y-BOCS	I.E., T	HAQ bond-related items	Mid	P	Yes	−0.43	−0.49 0.12	0.08 0.36	−0.51 −0.26
Keeley et al. (64)	25	44	M = 13.2 SD = 2.7	M = 10.48	EX/RP and CT	CY-BOCS	I.E.	TASC-R WAI	Early Mid	P T	Yes	−0.34	−1.11	−0.2	−0.7
Simpson et al. (58)	30	47	M = 39.9 SD = 13.4	M = 20.5	EX/RP or EX/MI	Y-BOCS	I.E.	WAI	Early	P T	Mediated by adherence	−0.39 −0.52	−0.85 −1.22	0.006 −0.16	−0.68 −0.76
Maher et al. (59)	28				EX/RP	Y-BOCS	I.E.	WAI	Early	P	Mediated by adherence				
Andersson et al. (65)	101	66	M = 34.9 SD = 12.7	M = 16.8 SD = 9	iCBT	Y-BOCS	I.E.	WAI	Mid	P	Yes	−0.14	0.28	0.06	−0.33
Wheaton et al. (66)	37	51	M = 33.8 SD = 12.5		EX/RP	Y-BOCS	I.E.	WAI	Early	P	Mediated by adherence	−0.1	−0.2	0.23	−0.41
Hagen et al. (67)	44P 13T	66	M = 23.7 SD = 9.7		EX/RP	Y-BOCS	I.E., T	WAI	Early	P	Yes	−0.36	−0.77	−0.1	−0.57
Herbst et al. (67)	30	65	M = 35	M = 34.8 SD = 137	iCBT	Y-BOCS OCI-R		WAI	Late		Yes	0.33	0.69	0.62	−0.03
Schwartz et al. (69)	155	60	M = 34.9 SD = 11.7		CBT with EX/RP	Y-BOCS	T, Self-rated	BPSR	Throughout	P	No				
Strauss et al. (70)	108 P 10 T	21	M = 4	M = 34.8 SD = 13.7	EX/RP-SMT	Y-BOCS OCI-R	I.E.	WAI	Different times	P T	Yes—EX/RP No—SMT				
Wolf et al. (71)	208 P 42 T	62	M = 35 SD = 10.2		EX/RP/ CBT/IBA	Y-BOCS	I.E.	WAI	Early	P T	Yes	−0.21	0.43	−0.07	−0.33

N, number of participants with obsessive-compulsive disorder; %F, percentage of females; EX/RP, Exposure with response prevention; CT, Cognitive Therapy; CBT, Cognitive-Behavioral Therapy; ICBT, Internet-based Cognitive Behavioral Therapy; MI, Motivational Interviewing; SMT, Stress Management Training; IBA, Inference Based Approach; MOCI, Maudsley Obsessional Compulsive inventory; Y-BOCS, Yale-Brown Obsessive-Compulsive Scale; CY-BOCS, Children’s Yale-Brown Obsessive-Compulsive Scale; OCI-R, Obsessive-Compulsive Inventory-Revised; P, Patient; T, Therapist; IE, Independent Evaluator; RI, Relationship Inventory; WAI-S, Working Alliance Inventory; HAq, Helping Alliance Questionnaire; TASC-R, Therapeutic Alliance Scale for Caregivers and Parents; BPSR, Bern Post-Session Report.

Moreover, all the articles were screened according to the research criteria for process research proposed by Lemmens et al. (45) and employed by Baier et al. (46) in a systematic review to assess the quality of the studies. These criteria comprised: (a) the use of a randomized controlled trial (RCT) design, (b) the use of a control group, (c) sample size defined as n ≥ 40, (d) the inclusion of different mediators in the design and statistical analysis, (e) assessment at two or more time points of alliance (not averaged during the analyses), and (f) experimental manipulation of the construct of the alliance.

The author F.S. performed a quality check and accuracy of the author’s V.S. data extraction. Inter-rater kappa reliability between the two coders was excellent (κ = 0.95), and minimal differences in coding were resolved through discussion till the agreement became perfect.

### Meta-analysis procedure

For the articles included in the systematic review, additional exclusion criteria were considered for conducting the meta-analysis in order to improve comparability between studies. Specifically, we included the studies that reported: (a) standardized β weights smaller than 0.5 or bigger than −0.5; (b) direct effect analysis of the relationship between the therapeutic relationship and outcome (see paragraph Statistical Analyses for more details).

### Statistical analyses

For each study, we extracted effect sizes computed as correlation coefficients or standardized β weights between therapeutic alliance and treatment outcome measures. Methodological considerations suggest that β weights should not be used as surrogates for correlation coefficients because they reflect the influence of the predictor variables in a multiple regression model (47). Thus, we approximated the bivariate Pearson correlation using the standardized regression coefficients as suggested by Peterson and Brown (48). The standardized β weights (that fell within an interval between −0.5 and 0.5) were transformed in Pearson correlation using the formula:


r=β+0.05⁢λ

where λ equals 1 when β is non-negative and 0 when β is negative.

The authors have shown that the relationship between *r* and β appears robust and independent of sample size and the number of predictor variables when within this interval. Indeed, they reported that “it is possible to derive a formula for imputing an *r* value missing assuming a knowledge of a corresponding β weight” because there is “a relatively tight joint distribution of β and *r* values within the range from −0.50 to 0.50” (48). To compute this formula we used the algorithm suited by the Practical Meta-Analysis Effect Size Calculator^4^ (49)].

In studies with more than one outcome measure, we averaged correlations or standard weights using the arithmetic mean to obtain one effect for each study, to avoid over-representing multi-analyses studies in the following analysis.

Then, each correlation was converted in Cohen’s *d* using the conversion software Psychometrica [Calculation of Effect Sizes,^5^ Dettelbach (Germany): Psychometrica]. By convention, an effect size of 0.2 is considered small, a value of 0.5 is moderate, and a value of 0.8 or greater is considered a relatively large effect (50).

The meta-analyses were performed with Jamovi 2 (MAJOR module following procedures suggested by Borenstein et al. (51) and Cooper (52). All analyses were carried out using the Fisher *r*-to-*z* transformed correlation coefficient as the outcome measure. We started by fitting a random-effects model to the data (53) and estimated the amount of heterogeneity (i.e., tau^2^) with the restricted maximum-likelihood estimator (54). In addition to the estimate of tau^2^, the *Q*-test for heterogeneity (55) and the *I*^2^ statistic were computed. In case any amount of heterogeneity was detected (i.e., tau^2^ > 0, regardless of the results of the *Q*-test), the software provided a prediction interval for the true outcomes.

To assess whether studies may be outliers and/or influential in the context of the model, we used studentized residuals and Cook’s distances (56). Studies with a studentized residual larger than the 100 × [1–0.05/(2 × k)]^th^ percentile of a standard normal distribution were considered potential outliers (i.e., using a Bonferroni correction with two-sided alpha = 0.05 for k studies included in the meta-analysis). Studies with a Cook’s distance larger than the median plus six times the interquartile range of the Cook’s distances are considered influential.

We computed the rank correlation test and the regression symmetry test to assess publication bias using the standard error of the observed outcomes as the predictor, and we created a funnel plot (57).

## Results

### Study selection

The literature search strategy and inclusion criteria yielded 847 studies that measured the relationship between the therapeutic alliance and post-treatment outcome in subjects with OCD. As shown in Figure 1, the literature search generated 802 potentially relevant articles (after 35 duplicates removal).

After titles and abstract screening, 763 were excluded. The full text of the remaining 39 eligible studies was retrieved and reviewed; articles were excluded either because they did not meet the inclusion criteria, or were qualitative studies, reviews, or commentaries.

This screening resulted in the inclusion of 13 articles for review. Studies were published between 1989 and 2022 and conducted in the United States or Europe. The sample sizes ranged from 17 to 208 for the patients and from 9 to 42 for the therapists. Most of the studies did not report the number of therapists who took part in the study. The average age ranged from 13 to 40 years old, and 55% were women. All articles reported that the participants had a diagnosis of OCD. In total, 897 patients took part in all studies reviewed [(58,59) employed the same sample of patients; thus, the sample was counted only once]. Eight studies reported the average age of onset, which varied from 16.81 to 34.8 years old.

Regarding the criteria for process research (45, 46), we note that seven studies (54%) were part of an RCT design in which different treatments were tested, and six had a control group. Eight studies (61%) had a sample size greater than 40 patients, and three (25%) employed multiple mediators in the experimental design. Finally, four studies (30%) assessed alliance more than in two-time points, and none did an experimental manipulation of the alliance between patient and therapist (Table 1).

Of the 13 selected articles, eight reported a relationship between therapeutic alliance and the outcome measure. Three studies found that patient adherence fully mediated the relationship, one found mixed results, and another did not find any relationship (Table 2).

**TABLE 2:** Studies meeting criteria for process research.

Study	RCT	Control group	*n* ≥ 40	Multiple mediators	Temporality	Manipulation
Hoogduin et al. (79)	0	0	1	0	0	0
Hoogduin et al. (79)	0	0	0	0	1	0
Keijsers et al. (63)	0	0	1	0	0	0
Vogel et al. (76)	0	0	0	0	0	0
Keeley et al. (64)	0	0	0	0	1	0
Simpson et al. (58)	1	1	0	1	0	0
Maher et al. (59)	1	1	0	1	0	0
Andersson et al. (65)	1	1	1	0	0	0
Wheaton et al. (66)	1	1	0	1	0	0
Hagen et al. (67)	0	0	1	0	0	0
Herbst et al. (68)	1	1	1	0	0	0
Schwartz et al. (69)	0	0	1	0	1	0
Strauss et al. (70)	1	1	1	0	1	0
Wolf et al. (71)	1	1	1	0	0	0

### Obsessive-compulsive disorder subtypes

Although the cardinal features of OCD are obsessions and compulsions, a variety of clinically significant obsessive-compulsive symptoms (e.g., checking, excessive washing, and ordering) may meet the diagnostic criteria for OCD. This pattern of heterogeneity that includes the age of onset (early vs. late onset), patterns of comorbidity, and presenting symptoms have been associated with different subtypes schemes presumably underlying different etiologies and neural correlates [e.g., (60–62)].

Only two articles provided information about OCD subtypes in the selected sample. Specifically, Maher et al. (59) reported that 4% of the sample belonged to the hoarding subtype; Keijsers et al. (63) reported that 53% of patients belonged to the checking subtype, 15% to the washing, 17% to the checking and washing, and 15% had obsessions only. However, no study investigated the relationship between the OCD subtype and alliance.

### Intervention/treatment

Most of the studies employed EX/RP intervention in various formats, from short and intensive [daily and lasting < 4 weeks; (64)] to more standard plans with weekly or twice-weekly sessions lasting between 4 and 8 weeks (58, 59, 65–71). Among these studies, four used a combined CBT protocol: the Pediatric OCD Treatment (64, 72), a CBT program that included both group and dyadic psychotherapy sessions (69), and the web-based ICBT (65, 68). All combined programs included a mix of psychoeducation, cognitive training, and EX/RP. Finally, one study employed also Stress Management Training [SMT; (73)] based on exposure with cessation of compulsions (58), one used the EX/RP augmented by motivational interviewing (MI) strategies (70), while another adopted the Inference Based Approach (IBA) (71).

Overall, all studies administered the EX/RP protocol either as a stand-alone treatment or in combination with other CBT interventions, and all patients received individual treatment.

### Outcome measurement

Symptom severity and treatment response were evaluated by the clinician-rated Yale-Brown Obsessive Compulsive Scale [Y-BOCS; (74, 75)], a semi-structured interview regarded as the “gold standard” in the measurements of OCD symptoms (obsessions and compulsions in the last week) (58, 59, 65–71, 76). Most of the studies employed independent evaluators for the rating, i.e., clinical psychologists, blind to treatment conditions and treatment outcomes. Only one study adopted both the clinician and self-report versions [Y-BOCS-SR; (69)]. Two studies also administered the Obsessive-Compulsive Inventory revised [OCI-R; 68, 70, 77)], and one used the Maudsley Obsessive-Compulsive Inventory [MOCI; (78)]. Both OCI and MOCI are self-report measures that assess the distress associated with obsessions and compulsions. Finally, only the earliest reviewed study adopted patients’ self-monitoring as an outcome measurement (79).

Despite some consistency in the scales used for outcome evaluation, studies considerably varied in the measurement timing. Two studies included only one time-point assessment at the end of the treatment (67, 68), two studies measured before and after the treatment (66, 71), six studies used three or four timepoints at baseline, mid-treatment, and at the end of the treatment (58, 59, 63, 64, 70, 76), and one study measured the symptoms with the self-report Y-BOCS at the end of each week (69). Finally, one study also took measurements at different time points during follow-up at 3, 6, 9, and 12 months (76). As it will be later discussed, the number of time points in which the outcome is measured represents an essential parameter for studying the reciprocal influence between symptom change and alliance.

Although symptom change represents an important parameter and a general index of treatment success, it might not be sufficient to depict the psychological wellness in the patient’s daily life. Indeed, an individual’s functioning presumably depends on both symptom severity and symptom management. Thus, assessing the quality of life in relation to therapeutic alliance and post-treatment outcomes seems to provide a complementary measurement. However, only one of the reviewed studies assessed the quality of life using the Quality of Life Enjoyment and Satisfaction Questionnaire [QLESQ; (80)], a self-report measure administered at different time points, and it did not find any relation between alliance and QLESQ (70).

Finally, as suggested by Buchholz and Abramowitz (32), another important variable to relate to the alliance is patients’ dropout during treatment. Recent studies seem to suggest a positive relationship between therapeutic alliance and patient retention [cf. review of (81–84)]. Despite its importance, only a few studies reported the number of patients that dropped the treatment, which was, in any case, a low rate.

### Alliance measurement

Five different measures of the therapeutic alliance, in varying formats, were used in these studies.

Most of the reviewed studies assessed the alliance with the Working Alliance Inventory [WAI; (85)], a self-report inventory that was originally designed to measure Bordin’s working alliance dimensions (bond, task, and goals). Five studies administered the patient-rated version (58, 59, 65–67), three used both the patient and therapist versions (64, 70, 71), and one did not specify which version was used (68). Among these studies, some employed the standard version composed of 36 items (58, 59, 64), while others adopted the short forms composed of 12 items by Tracey and Kokotovic (65, 67, 68, 70, 86) or by Hatcher and Gillaspy (66, 68, 87). Keeley et al. (64), that assessed the therapeutic alliance in a pediatric population, also administered the WAI to the caregivers and the Therapeutic Alliance Scale for Children-Revised to the patients [TASC-R; (88)].

The remaining studies assessed the quality of alliance using the self-report Barret-Lennard Relationship Inventory [RI, (89)] (79), the items related to the bond of the Helping Alliance Questionnaire HAq (90, 76), and the alliance subscale of the Bern Post-Session Report (91). These scales differ from the WAI in several aspects, such as the dimensions of the alliance that are represented and the number of items that the measures contain. For instance, the RI, a measure of empathy, correlates only with the WAI bond scale but not with the task and goals dimensions (44). Meta-analytic results have shown that the different versions of the same scale (e.g., long or short format and different rater versions) differed in predicting treatment outcomes (30). Despite some similarities and shared themes between the alliance scales, they do not have a common account of the alliance construct (92, 93). In particular, no scale has a complete representation of the different properties belonging to the concept of alliance that has been proposed in the past years.

Another potential confounding factor in the alliance-outcome relationship is the variability across alliance raters. Ideally, a good measure of the alliance should have a good consistency, measured as inter-rater reliability (94). Horvath et al. (30) estimated that the variables “type of measure” and “raters” account for 23% of the variance in predicting the treatment outcome. However, the variability across raters *per se* does not seem to represent a strong methodological issue, given the moderate correlation between patients’ and therapists’ alliance scores (31). Nevertheless, the reviewed studies showed mixed results in predicting treatment outcomes among alliance raters. For instance, Keeley et al. (64) and Hoogduin et al. (79) found that only therapist rating predicted treatment outcome when the alliance was measured in an early stage. However, both therapist and patient predicted the treatment outcome when the alliance was measured in a mid-phase. Conversely, Strauss et al. (70) found that only patient averaged alliance scores covaried with outcome treatment, but patient and therapist early scores were not associated with symptom change. Some mixed results were also shown in the study published by Keijsers et al. (63), where patient ratings correlated with a reduction in obsessive fear; however, only therapist ratings classified patients as success or treatment failure with multivariate analysis. Finally, Wolf et al. (71) showed that only therapist rating measured in an early phase significantly predicted the post-treatment outcome. Taken together, these results show that therapist and patient ratings differed in how they predicted treatment outcomes across studies. Thus, it would be helpful to relate these differences across raters in predicting symptom change with some inter-rater reliability measure (95). Indeed, only one study reported a measure of consistency across raters and found a weak significant correlation (71), although most recent studies reported overall good reliability of alliance scores measured with Cronbach’s alpha.

Albeit, the therapeutic alliance has been described as an intrinsic dyadic concept that involves a process of mutual influence and impact between the therapist and the patient [e.g., (23, 96–98)], 50% of the studies reviewed here asked only patients to rate alliance. This limitation reflects the main trend in therapeutic alliance studies that mostly focus on patients’ views only (30). However, recent studies have highlighted the importance of taking into account both patient and therapist perspectives, introducing the concept of patient-therapist alliance “congruence” (i.e., the inter-rater agreement on alliance quality at one time-point) and the alliance “convergence” (i.e., the degree of change over time in the inter-rater agreement on alliance quality) (99–101). These constructs reflect the dynamic nature of this dyadic process and carry complementary information on a therapeutic relationship that is asymmetric by nature (102).

Wide variability across studies was also observed in the evaluation timing. Indeed, seven studies collected data only at one time-point, either in the early (58, 59, 63, 66, 67, 71, 76), mid (76), or late phase of the treatment (68). Two studies used three or four time-points at baseline, mid-treatment, and at the end of the treatment (64, 70), and one study measured alliance through all treatment at the end of each week (69).

### Alliance-outcome relationship measurement

All studies reviewed here assessed the relationship between alliance and some measure of symptom outcome. The two earliest studies computed the correlation between alliance and self-report symptom outcome (79) or obsessive fear symptom change (63). Both studies found evidence of a positive effect of alliance on the treatment outcome. However, these results should be taken with caution because of the less rigorous methodology employed.

Subsequent studies investigated the role of the alliance as a mediator of symptom change with more sophisticated statistical analyses, such as a causal stepwise approach using linear regressions (103) and structural equation modeling (104). Other methods included growth analysis (105) and the longitudinal mixed-effects model (106). Overall, the nine studies that used a regression approach found evidence of alliance as a predictor of positive change. However, three of eight found that the general effect of alliance [(58, 59), with an overlapping sample] or task alliance (66) on treatment outcome was fully mediated by patient’s adherence to between- and within-session exposure tasks. Among the most recent studies having the highest frequency of ratings of both alliance and symptoms (i.e., at the end of each week or throughout all treatment), no alliance’s influence on symptom decrease was found (69, 70). The only study that compared simultaneous and cross-lag models to assess the effect of reciprocal influence in the alliance-outcome relationship found that patient alliance covaried with symptoms change as measured with the Y-BOCS; however, changes in previous Y-BOCS scores predicted subsequent changes in alliance scores, thus suggesting that an improvement in the reported symptoms precedes the changes in the alliance scores (70).

Furthermore, among the studies that assessed alliance on patients alone, one followed the data analysis suggested by Baldwin et al. (107). This analysis consists of decomposing alliance-outcome correlations into two components: the “within-therapist correlations” at the patient level (i.e., how the alliance is related to outcome in each therapist) and “between-therapist correlations” at the therapist level (i.e., how the alliance is related to outcome across therapists). Importantly, this method allows computing the cross-level interaction between patients’ and therapists’ variability (107). In the study that implemented this analytic method, it was found that therapist variability in the task/goal dimension of the alliance predicted treatment outcome, while patient variability in the alliance did not. Conversely, the therapeutic bond was not related to the outcome.

Overall, this qualitative analysis of the reviewed studies points to some indication of a positive relationship between alliance and treatment outcome. Nevertheless, given the high degree of methodological variance even in the most recent studies that employed more advanced statistical and experimental design, these results should be interpreted in the context of this variability across studies.

### Meta-analysis

The purpose of this section of the review is to quantify the present literature regarding the existence and strength of the relation between therapeutic alliance and outcome in the context of OCD. As the preceding qualitative analysis illustrated, there is considerable variability across studies with regard to how and when alliance and outcome were measured, who were the raters, and how the relationship alliance-outcome was measured. Thus, to improve comparability among the results, some exclusion criteria were applied that narrowed down to k = 6 the number of studies that were included in the analyses (64–67, 71, 76). In particular, one study was excluded because it included self-monitoring as outcome measure and did not use a validated alliance scale (79); the others did not report any statistics that could be used in the meta-analysis (59, 63, 68–70, 73). All the pooled effect sizes reflected patient’s rating.

**FIGURE 2:**
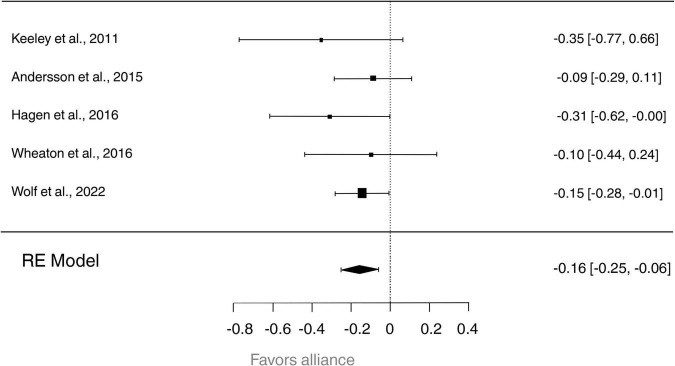
Forest plot visualizing the relationship between the global therapeutic alliance and the treatment outcome for each included study. Horizontal bars show 99% confidence intervals, with the study having a significant effect denoted by horizontal bars that do not touch the dotted vertical line (the line of no effect). Diamonds sizes reflect the weight of the overall study.

We first performed a model with the global alliance score (task, goal, and bond). In the selected studies (64–67, 71), the observed Fisher *r*-to-*z* transformed correlation coefficients ranged from −0.3541 to −0.0902, with most estimates being negative (100%). The estimated average Fisher *r*-to-*z* transformed correlation coefficient based on the random-effects model was *Tau*^2^ = −0.1562 (*C.I.95%*: −0.2542 to −0.0582; Figure 2). Therefore, the average outcome differed significantly from zero (*z* = −3.1231, *p* = 0.0018). According to the *Q*-test, there was no significant amount of heterogeneity in the true outcomes [*Q*(4) = 2.3834, *p* = 0.6656, *tau*^2^ = 0.0000, *I*^2^ = 0.0000%]. Hence, even though there may be some heterogeneity, the true outcomes of the studies were generally in the same direction as the estimated average outcome.

**FIGURE 3:**
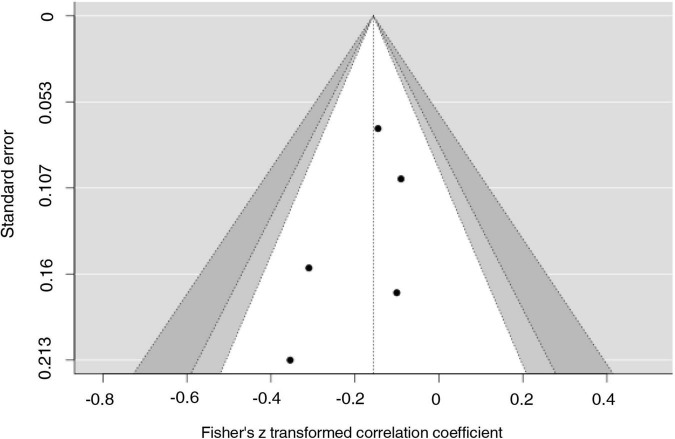
Funnel plot. Estimates (*z*-score) from selected studies (on the horizontal axes) plotted against each study’s standard error (on the vertical axes).

An examination of the studentized residuals revealed that none of the studies had a value larger than ± 2.5758; hence there was no indication of outliers in the context of this model. According to Cook’s distances, none of the studies could be considered overly influential. Neither the rank correlation nor the regression test indicated any funnel plot asymmetry (*p* = 0.8167 and *p* = 0.3780, respectively; see Figure 3).

Next, we pooled the effect sizes for the task/goal alliance dimension and for the bond dimension separately. For the task/goal dimension, in the included studies (66, 67, 71), the transformed correlation coefficient based on the random-effects model was *Tau*^2^ = −0.1977 (95% CI: −0.3149 to −0.0805). Therefore, the average outcome differed significantly from zero (*z* = −3.3071, *p* = 0.0009; Supplementary Figure 1). According to the *Q*-test, there was no significant amount of heterogeneity in the true outcomes [*Q*(2) = 1.9141, *p* = 0.3840, *tau*^2^ = 0.0000, *I*^2^ = 0.0244%].

For the bond dimension, in the included studies (66, 67, 71, 76), the transformed correlation coefficient based on the random-effects model was *Tau*^2^ = −0.1372 (95% CI: −0.3669 to −0.0924). Therefore, the average outcome did not differ significantly from zero (*z* = −1.1710, *p* = 0.2416; Supplementary Figure 2). According to the *Q*-test, the true outcomes appear to be heterogeneous [*Q*(3) = 9.2209, *p* = 0.0417, *tau*^2^ = 0.0351, *I*^2^ = 66.2000%]. A 95% prediction interval for the true outcomes is given by −0.5703 to 0.2958. Hence, although the average outcome is estimated to be negative, in some studies the true outcome may in fact be positive. This amount of heterogeneity might depend on pooling effect sizes from two different scales (WAI and HAq). Although both scales measure the bond dimension and are presumably correlated, they cannot be traced back to a common second-order factor.

## Discussion

This systematic review aimed to synthesize the current scientific evidence on the relationship between therapeutic alliance and treatment outcome in CBT in patients with OCD.

Overall, we found a modest association between alliance and treatment outcome. This result is consistent with previous systematic reviews and meta-analyses that quantified this relationship with an aggregate *r* of 0.28 across disorders and treatments (27) or found a positive association between alliance and symptom change in anxiety-related disorders (32, 33). In particular, our results suggest that task and goal alliance domains, which may be more characterized as cognitive factors (32), are associated with post-treatment outcomes, while the therapeutic bond is not. Most of the included studies measured alliance in the early or mid-phase of the treatment. Thus, it is possible that agreeing on the goals and the willingness to be engaged in the exposure tasks might be predictive factors in the first sessions of the therapy.

We also found substantial variability of different sources across studies. Hoogduin et al. (79) and Keeley et al. (64) reported that both early- and mid-treatment therapist alliance, but only mid-treatment patient alliance, was associated with an improved outcome in adult and pediatric populations, respectively. Consistently, Vogel et al. (76) and Andersson et al. (65) reported that mid patient alliance was associated with better outcomes. Conversely, Keijsers et al. (63) found that early patient alliance but not therapist alliance was associated with improvement in obsessive fears (though not to compulsive behaviors). Similarly, Hagen et al. (67) found that early patient task and goal alliance (though not bond) was associated with a better outcome, while Wheaton found that only early patient task alliance predicted post-treatment outcome. Also, Wolf et al. (71) found that early task and goal alliance and early therapist alliance (total score) predicted the post-treatment outcome. Differently from these studies that reported a positive association between alliance and outcome, one study found that overall early patient alliance was not related to outcome (69). Moreover, one of the most recent studies found a mixed pattern of results, and only in EX/RP (not in SMT) symptom change was associated with subsequent changes in alliance (though not vice versa) (70). Finally, three studies found that overall early patient alliance was mediated by adherence (58, 59, 66).

All the reviewed studies differ in many methodological aspects, such as outcome and alliance measurements, alliance raters and timing, and statistical approaches. Most recent studies which adopted more sophisticated analyses (e.g., structural equation, linear growth analyses, stepwise regression analysis, and cross-lagged model), took into account early symptom improvement and examined temporal associations of the alliance over time, are also showing less or no evidence of alliance affecting treatment outcome. Thus, our results should be interpreted in the context of this variability, and several limitations should be considered. For instance, half of the studies employed RCTs to examine differences across treatment, and used a control group. Second, 58% of studies used a sample size equal to or greater than 40, that is generally considered the minimum number to have sufficient power in the meta-analysis (108, 109). However, our meta-analysis, which had an average sample size of 66 participants, is sufficiently powered to detect small effects [1-β err prob 0.99, formula retrieved by (110)]. Third, three studies disentangled the effect of alliance from other factors, such as symptom severity, adherence to treatment, quality of life, patient’s expectancy, and motivation, and OCD sub-types (e.g., washing, hoarding). Therefore, more mediation studies are needed to assess how alliance contributes to mediate the relationship between treatment and symptom change and to separate its effect from other specific and generic factors (e.g., age, gender, comorbidity with other disorders, age of onset, use of psychiatric medication, patient’s and therapist’s factors). Fourth, the small number of available studies and the fact that some of them were carried out by some overlapping research groups (and presumably with similar programs and methodology), although mostly with different samples, makes it difficult to reach firm conclusions. Fifth, only four studies assessed alliance at two or more time-points, not averaging during the analyses (64, 69, 70, 79).

Temporality represents an essential parameter in experimental designs investigating the therapeutic alliance. While in the past, process-based research tended to represent the association between alliance and outcome as a static “snapshot” by taking measurements at only one time-point or by averaging, the inherently dynamic nature of the therapeutic interaction is now being increasingly recognized [e.g., (111)]. This change of view, which implies studying how alliance changes over the course of treatment through cycles of ruptures and repairs (112), and how it interacts with the specific treatment, entails achieving a fuller picture of this dynamic dyadic process. Indeed, a key factor in studying this phenomenon implies understanding the direction of the alliance-outcome link. A positive link might have at least three different sources: (a) alliance produces patient’s symptoms change; (b) symptom change induces a change in the therapeutic alliance between therapist and patient; (c) alliance and treatment outcome influence each other (113). To test the idea that alliance influences treatment outcome, the alliance must predict outcome as measured at a subsequent time point, taking into account potential changes in the outcome preceding alliance measurement (114). Thus, a gold standard for future studies would be to collect repeated measurements of alliance and outcome (after and during session) and combine them with more sophisticated statistical approaches (e.g., cross-lagged panel model) to analyze the interactions and reciprocal influences between these variables over time. Among the reviewed studies Schwartz et al. (69), had the highest sampling rate, assessing alliance and outcome at the end of each session/week. On the other hand Strauss et al. (70), compared simultaneous and cross-lagged models to study the reciprocal influences between alliance and outcome but sampling fewer time-points.

Another important factor to consider is the different impact of therapeutic alliance domains on symptoms change. Indeed, three studies that disentangled the effect of goal, task and bond, found that treatment outcome was significantly predicted by goal and task alliance but not by bond (66, 67, 71). This result could be associated with the specific EX/RP techniques that are used for the treatment of OCD, that require setting appropriate goals, and providing tasks that allow patients to go far enough in exposures to the fearful stimuli or situations (115). It is possible to argue that such a structured and challenging treatment protocol, for both therapist and patient, creates the basis for building an alliance that relies more on tasks and goals consensus than on emotional attachment and more in general on the feelings and attitudes that patient and therapist have toward each other. It is also possible to speculate that bond might represent a more significant outcome predictor in therapies with less structured protocols.

Finally, it must be noted that a significant association between alliance and outcome does not imply a significant clinical impact. For instance, Wolf et al. (71) found that although therapeutic alliance significantly predicted post-treatment outcome, it accounted for only 2% of symptoms improvement, resulting in alliance being not clinically relevant in their sample. This result might be due to the high rates in the alliance scores that makes it difficult to reach higher scores and thus a stronger association between alliance and outcome (71). Future studies need to take into account alliance scores variability and possible ceiling effects that might hinder the effect of alliance on post-treatment outcome.

Some limitations should be considered when interpreting these results. First, in this review, there is a possible language bias since the research strategy was limited to articles published in English, and we also did not include unpublished studies. Therefore, it is possible that some relevant papers were missed. Also, since studies with samples of different ages were included, it was not possible to draw conclusions on specific populations. Moreover, most of the studies in the current review were based on research protocols that included structured interventions and highly trained therapists; thus, the generalizability of these results to more naturalistic settings (e.g., general community practice and patients with comorbidities) remains unclear. Naturalistic studies are required to demonstrate whether alliance works in the field with patients with OCD. Finally, for the statistical analyses, we used the approach proposed by Peterson and Brown (48) to convert the standardized β weights into Pearson correlation coefficients. Although this approach has been widely used in the literature because it provides a straightforward method to deal with missing values, it has some limitations. Recently, it has been shown that this approach can lead to an underestimation of meta-analytic correlations and that the estimated correlations do not perform better than using existing correlations (116, 117). Thus, our meta-analytic results should be interpreted in the context of this trade-off between generalization and approximation.

In summary, the present review shows some evidence of an interactive effect between alliance and the treatment outcome in individuals with OCD, although with considerable variability in reporting varying measurement time-points, experimental designs, statistical approaches, measurements tools, and alliance raters. However, we sought to achieve the greatest possible consistency in our data extraction and meta-analysis.

Future studies that include more refined temporal assessment of alliance, larger samples, and measures of potentially interacting variables are required to better understand whether changes in alliance interact with treatment response or vice versa and which is the clinical impact of this association. Understanding this complex relationship will ultimately help to improve outcomes for individuals living with OCD.

## Data availability statement

The original contributions presented in this study are included in the article/Supplementary material, further inquiries can be directed to the corresponding author/s.

## Author contributions

AMS, GG, and FM conceived the study. FS and VS reviewed the literature, analyzed the data, and wrote the first version of the draft. GA supervised the statistical analyses. GD’A, TD, and NP contributed to the discussion of the literature. All authors contributed to the writing and editing of the draft.

## Footnotes

1
https://www.bmj.com/content/372/bmj.n71

2
https://osf.io/dxez5/

3
https://www.mendeley.com/reference-management/reference-manager

4
https://www.campbellcollaboration.org/escalc/html/EffectSizeCalculator-SMD22.php

5
https://www.psychometrica.de/effect_size.html
