# Supplementary material for: The therapeutic alliance in cognitive-behavioral therapy for obsessive-compulsive disorder: A systematic review and meta-analysis
Source: Front Psychiatry. 2022 Sep 6;13:951925. doi: 10.3389/fpsyt.2022.951925 (PMC9488733; doi:10.3389/fpsyt.2022.951925)
Supplement: Supplementary file 1 [file Data_Sheet_1.docx]

**Supplementary Information**

**
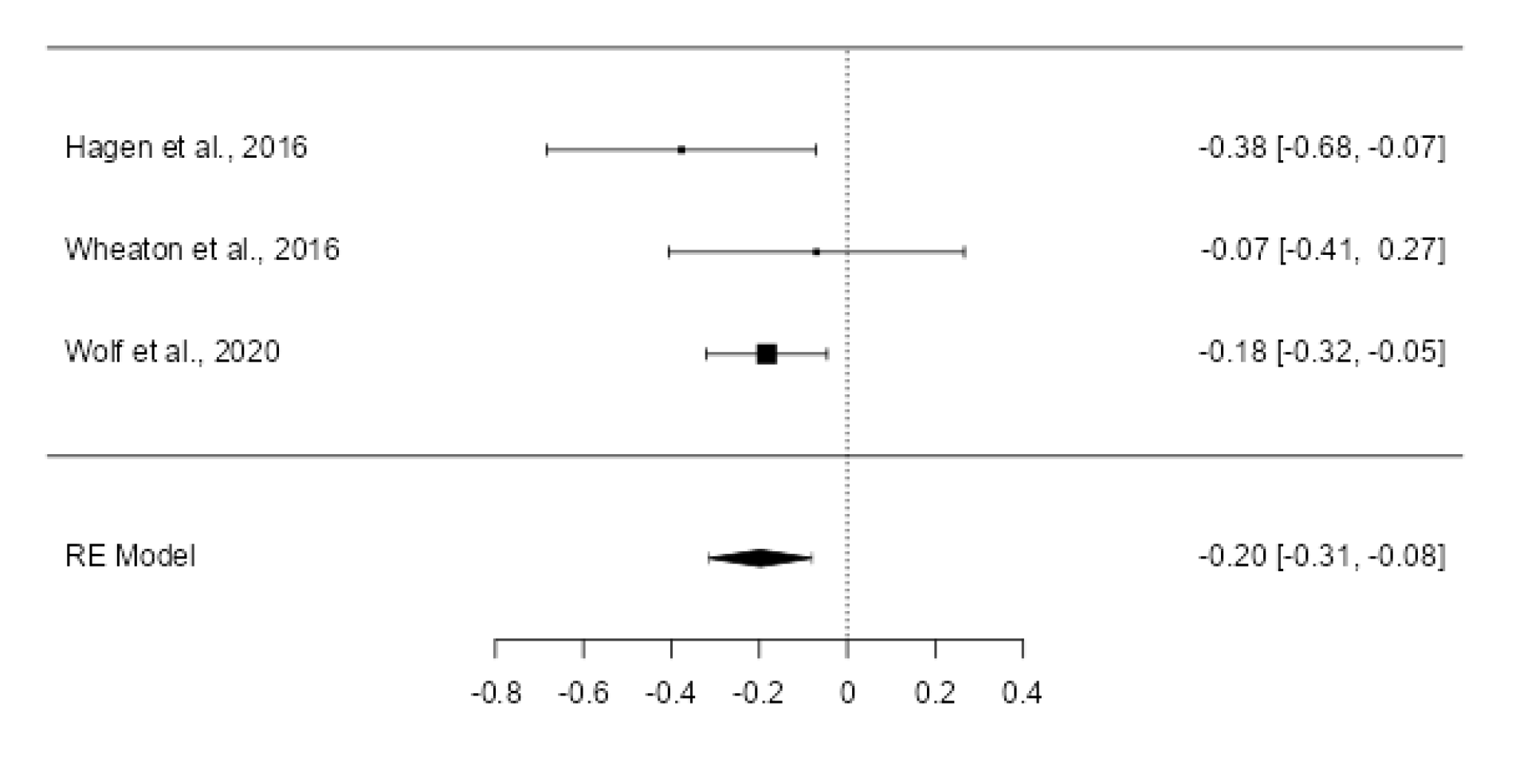
**

**Supplementary Figure 1. The forest plot visualizes the relationship between the task/goal therapeutic alliance and the treatment outcome for each included study.** Horizontal bars show 99% confidence intervals, with the study having a significant effect denoted by horizontal bars that do not touch the dotted vertical line (the line of no effect). Diamond sizes reflect the weight of the overall study**.**

**
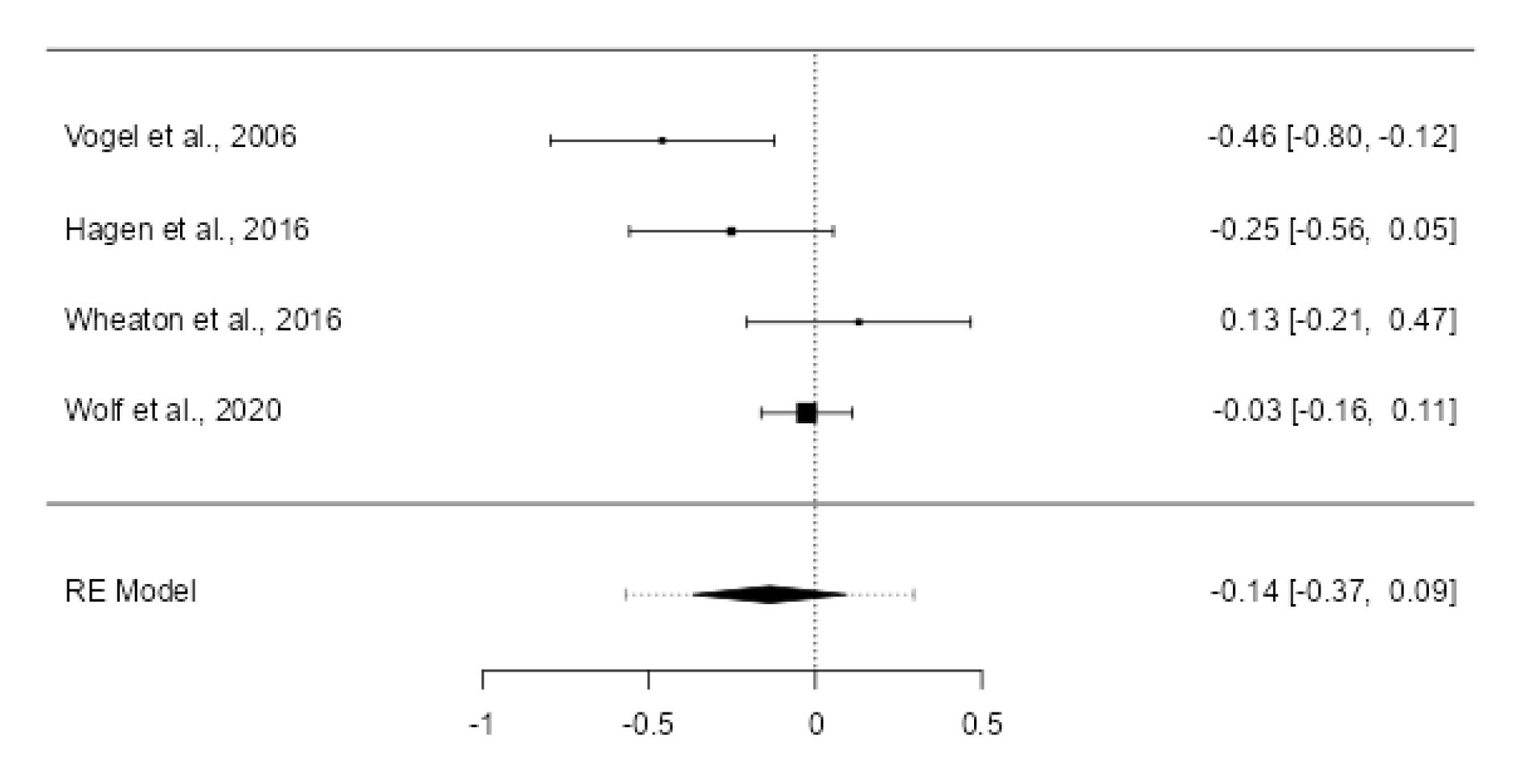
**

**Supplementary Figure 2. The forest plot visualizes the relationship between the bond therapeutic alliance and the treatment outcome for each included study.** Horizontal bars show 99% confidence intervals, with the study having a significant effect denoted by horizontal bars that do not touch the dotted vertical line (the line of no effect). Diamond sizes reflect the weight of the overall study**.**
